# Central Vein Sign and Paramagnetic Rim Lesions: Susceptibility Changes in Brain Tissues and Their Implications for the Study of Multiple Sclerosis Pathology

**DOI:** 10.3390/diagnostics14131362

**Published:** 2024-06-27

**Authors:** Carolina de Medeiros Rimkus, Fábio Seiji Otsuka, Douglas Mendes Nunes, Khallil Taverna Chaim, Maria Concepción Garcia Otaduy

**Affiliations:** 1 Department of Radiology and Oncology, Hospital das Clínicas da Faculdade de Medicina da Universidade de Sao Paulo (HCFMUSP), Sao Paulo 05403-010, SP, Brazil; douglas.nunes@hc.fm.usp.br (D.M.N.); maria.otaduy@hc.fm.usp.br (M.C.G.O.); 2 Laboratory of Medical Investigation in Magnetic Resonance-44 (LIM 44), University of Sao Paulo, Sao Paulo 05403-000, SP, Brazil; fabio.otsuka@usp.br (F.S.O.); khallil.chaim@hc.fm.usp.br (K.T.C.); 3 MS Center Amsterdam, Anatomy and Neurosciences, Vrije Universiteit Amsterdam, Amsterdam UMC, Location VUmc, 1081 HV Amsterdam, The Netherlands; 4 Instituto D’Or de Ensino e Pesquisa (IDOR), Sao Paulo 01401-002, SP, Brazil; 5 Grupo Fleury, Sao Paulo 04701-200, SP, Brazil

**Keywords:** susceptibility-weighted image, multiple sclerosis, paramagnetic rim lesion, central vein sign, magnetic susceptibility, chronic active lesion

## Abstract

Multiple sclerosis (MS) is the most common acquired inflammatory and demyelinating disease in adults. The conventional diagnostic of MS and the follow-up of inflammatory activity is based on the detection of hyperintense foci in T2 and fluid-attenuated inversion recovery (FLAIR) magnetic resonance imaging (MRI) and lesions with brain–blood barrier (BBB) disruption in the central nervous system (CNS) parenchyma. However, T2/FLAIR hyperintense lesions are not specific to MS and the MS pathology and inflammatory processes go far beyond focal lesions and can be independent of BBB disruption. MRI techniques based on the magnetic susceptibility properties of the tissue, such as T2*, susceptibility-weighted images (SWI), and quantitative susceptibility mapping (QSM) offer tools for advanced MS diagnostic, follow-up, and the assessment of more detailed features of MS dynamic pathology. Susceptibility-weighted techniques are sensitive to the paramagnetic components of biological tissues, such as deoxyhemoglobin. This capability enables the visualization of brain parenchymal veins. Consequently, it presents an opportunity to identify veins within the core of multiple sclerosis (MS) lesions, thereby affirming their venocentric characteristics. This advancement significantly enhances the accuracy of the differential diagnostic process. Another important paramagnetic component in biological tissues is iron. In MS, the dynamic trafficking of iron between different cells, such as oligodendrocytes, astrocytes, and microglia, enables the study of different stages of demyelination and remyelination. Furthermore, the accumulation of iron in activated microglia serves as an indicator of latent inflammatory activity in chronic MS lesions, termed paramagnetic rim lesions (PRLs). PRLs have been correlated with disease progression and degenerative processes, underscoring their significance in MS pathology. This review will elucidate the underlying physical principles of magnetic susceptibility and their implications for the formation and interpretation of T2*, SWI, and QSM sequences. Additionally, it will explore their applications in multiple sclerosis (MS), particularly in detecting the central vein sign (CVS) and PRLs, and assessing iron metabolism. Furthermore, the review will discuss their role in advancing early and precise MS diagnosis and prognostic evaluation, as well as their utility in studying chronic active inflammation and degenerative processes.

## 1. Introduction

Multiple sclerosis (MS) is a chronic inflammatory and demyelinating disease of the central nervous system (CNS). MS-lesions have a distinct shape and growth around CNS veins. However, they might have a similar appearance to other CNS-inflammatory and non-inflammatory diseases, especially in conventional magnetic resonance imaging (MRI) sequences, such as Dual-echo, T2-weighted, and T2-FLAIR images, on which non-MS lesions appear as the focal areas of hyperintensity [1]. The new revisions of the McDonald criteria for MS-diagnostic emphasize the need for a more sensitive and earlier diagnostic of MS. However, a higher sensitivity cannot imply a lower specificity. Thus, it is important to exclude alternative diagnostic and MS-mimics [2]. In this scenario, susceptibility-weighted imaging (SWI), with its capability to detect the central vein within a typical MS-lesion, emerges as a promising tool to boost the specificity of MS-diagnostic [3,4,5].

Although the formation of MS-plaques is associated with blood–brain barrier (BBB) disruption, the inflammatory process persists in the CNS “behind” a closed or repaired BBB, as a compartmentalized inflammation. Importantly, the compartmentalized inflammation might still happen in the absence of apparent new lesion formation or BBB disruption, affecting the normal-appearing brain tissues (NABT), the cortical gray matter, and the meninges [6].

Conventional T2-FLAIR and T1-gadolinium-enhanced MRI sequences are used to capture the detectable radiological “inflammatory” activity [2] that manifests as new/growing macroscopic lesions and signs of BBB disruption [7]. However, they are not sufficient to capture the whole MS-pathology [8]. SWI is an MRI technique sensitive to paramagnetic substances, offering a distinct advantage in the study of detailed MS-pathology [9].

SWI is sensitive to paramagnetic substances, such as iron, allowing for the precise visualization of the iron rim associated with activated microglia [10]. This enhanced capability enables clinicians and researchers to non-invasively monitor microglia activity, being valuable to depict latent inflammation within MS lesions [11,12] and NABT [13]. Dynamic changes in iron metabolism are also useful to detect oxidative injury and subsequent mitochondrial damage, which might play a role in chronic inflammatory activity and MS-degenerative processes [14,15].

In this review, we describe the physical principles of susceptibility-weighted techniques, including T2*-weighted images, the components of SWI, and the new information added by quantitative susceptibility mapping (QSM). Our focus is to discuss their use in MS, especially in the detection of the central vein sign, iron rim lesions, and the assessment of the iron metabolism, along with their implications for early and precise MS-diagnostic, prognostic value, the study of chronic active inflammation and degenerative processes.

## 2. Physical Principles

### 2.1. Magnetic Susceptibility

Magnetic susceptibility (χ) is a physical property that describes the relationship between the magnetic response of a substance due to an applied magnetic field, i.e., the magnetic field strength of the MRI scanner (*B*_0_). A compound’s magnetic susceptibility can be understood as a collective behavior of the many electrons in a given sample, whose arrangement has an impact on the resulting magnetic field. Magnetic susceptibility is a dimensionless property and given its small number (×10^−6^) it is commonly expressed in parts per million (ppm) or in parts per billion (ppb). 

Variations in susceptibility within the brain tissue (Δχ) will lead to variations in the magnetic field strength (Δ*B*), as given by the following equation [16], which in turn will affect the MRI signal:

$$\Delta B=\Delta \chi B_{0}$$

In a biological context, substances can be classified into two magnetic groups: paramagnetic and diamagnetic substances. Diamagnetic substances are characterized by a negative magnetic susceptibility χ, leading to a slight decrease in the net magnetic field strength. Water [17], lipids, myelin, calcium, and many proteins are diamagnetic [18,19,20], which makes the biological tissue predominantly diamagnetic [21]. On the other hand, paramagnetic substances are characterized by a positive magnetic susceptibility χ, which will alter the precession frequency of the MRI signal in the opposite direction of diamagnetic substances [19,22]. In general, substances containing unpaired electrons in their structures, such as iron and copper, have a paramagnetic property [21]. 

**Figure 1:**
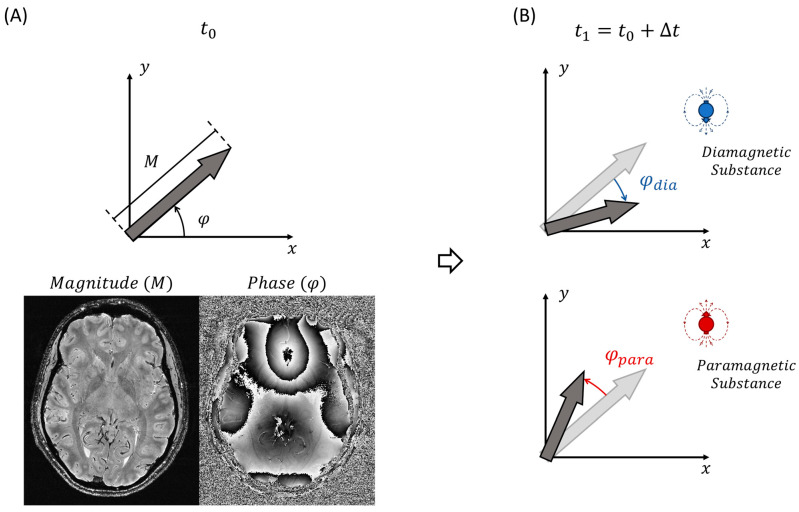
**Magnetic susceptibility decay.** (**A**) Representation in the xy plane of the magnetization vector of a single voxel at the initial time t0, the vector is described by its magnitude (M) and phase (φ) relative to the reference axis (x-axis in this representation), below is the representation of the magnitude and phase images of an axial brain slice. (**B**) After a time ∆t has passed, the magnetization vector will evolve differently depending on the local magnetic field caused by its neighbors. In the case of a diamagnetic surrounding (represented by the blue dipole), the magnetization will rotate in a clockwise direction, while in the case of a paramagnetic surrounding (represented by the red dipole), the magnetization will rotate in a counterclockwise direction. Notice that in both cases, the magnitude of the magnetization decays.

The presence of substances with different χ within the tissue will have two main impacts on the measured MRI signal: (1) a decrease in the magnitude component, due to the increase in the transverse relaxation rate, which can be appreciated as hypointensities on T2 and T2*-weighted images, or hyperintensities on R2 and R2* maps, and (2) a change in the phase of the signal, which can be either positive or negative depending on the sign of the bulk magnetic susceptibility in the voxel [22,23]. Figure 1A illustrates the magnitude and phase component of the MRI signal.

Table 1 lists the absolute magnetic susceptibility χ of some biological compounds commonly found in the human brain and the net magnetic susceptibility of some brain structures in the healthy brain and in MS lesion.

### 2.2. Susceptibility-Weighted Techniques 

The complex signal of the MRI of a voxel is related to transverse magnetization, which can be understood as the vector sum of all hydrogen proton’s magnetic moments inside the voxel. The intensity of the signal is related to the magnitude of the magnetization vector, and the accumulated angle relative to a reference axis is related to its phase (Figure 1).

**Figure 2:**
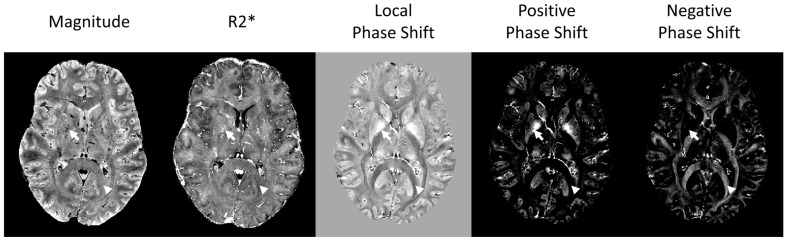
Example of magnitude, R2* map, and local phase shift images of a healthy subject. Only in the phase shift image is it possible to distinguish between paramagnetic (arrow) and diamagnetic (arrowhead). As evidenced by the figures on the right, paramagnetic compounds have a positive phase shift and diamagnetic compounds have a negative phase shift.

In a gradient echo sequence (GRE), both the magnitude and phase of the MRI signal are affected by the magnetic susceptibility. The magnitude decays over time due to T2* effects which are associated both with the mobility of the spins (T2) and magnetic susceptibility distortions (T2′) [26]. Therefore, hypointense signals on T2*-weighted images denote the presence of substances with different susceptibility to water (the main component in the tissue). In order to be more quantitative, R2* maps (whereby R2* = 1/T2*) [23] can be obtained by acquiring a multi-echo gradient echo sequence (usually around 5–10 echoes with TEs ranging from the possible minimum value to about 30–50 ms, whereby this value decreases with increasing scanner field strength) and by applying a simple monoexponential function to the observed signal decay as a function of TE. This postprocessing is incorporated in most scanners, which automatically produce R2* maps. The choice of R2* maps instead of T2*maps is merely practical, because on R2* maps the foci of different susceptibility will appear bright (hyperintense), which are easier to identify. On the other hand, the accumulated phase is shifted relative to a reference axis and depends on the net magnetic susceptibility effect of its neighbors, such that, if the net magnetic susceptibility is diamagnetic, the phase will evolve in a clockwise direction, and if it is paramagnetic then the phase will evolve in a counterclockwise direction (Figure 1B). Therefore, while the magnitude information does not distinguish between diamagnetic and paramagnetic effects, the phase evolution over time is able to differentiate these effects. Whether diamagnetic substances will appear bright or dark on phase images will depend on the direction of the main static magnetic field in the scanner, i.e., whether it is a right- or left-handed system. In left-handed systems, the phase of paramagnetic substances will have a bright signal, while high diamagnetic substances will have a dark signal [9,27]. In a right-handed system, this behavior will be the opposite. Figure 2 shows an example of a magnitude image, an R2* map, and a high-pass filtered phase image; only the phase image allows the distinction between diamagnetic and paramagnetic tissues. 

QSM and SWI are MRI techniques that take advantage of the phase information of the GRE’s complex signal [28,29] to produce the resulting image. The underlying mechanism of contrast in these techniques is the bulk magnetic susceptibility of the tissue, which makes these techniques highly sensitive to magnetic composition variations in the tissue.

Although both SWI and QSM rely on the same physical principle, they represent different aspects of magnetic susceptibility. SWI is a qualitative map, meaning that its values cannot be used for a direct comparison between subjects, scans, or different sites, but it is more practical since only the acquisition of one echo (one TE) is required. Meanwhile, QSM offers all the advantages of a quantitative technique but requires the acquisition of multiple echo times and sophisticated offline post-processing.

**Figure 3:**
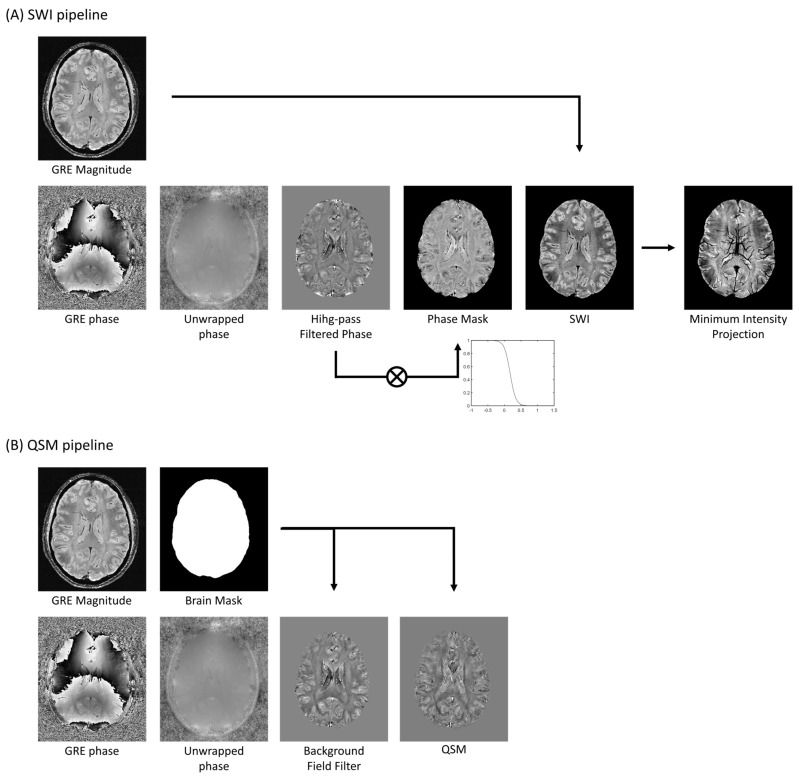
**Processing pipelines.** (**A**) SWI pipeline processing. Phase image is first unwrapped in order to remove phase wraps; the unwrapped phase is then high-pass filtered to remove non-tissue related phase shifts; the filtered phase is then turned into a phase mask, in this example a sigmoid function was applied; by multiplying the magnitude image with the phase mask the SWI is generated. Furthermore, a minimum intensity projection can be used to enhance the veins. (**B**) QSM pipeline processing. Phase is first unwrapped and then background field contributions are removed using dedicated algorithms. Finally, the dipole inversion is applied in order to retrieve the QSM maps.

SWI-phase images are high-pass filtered to remove phase contributions from sources non-related to the tissue, such as background fields and field inhomogeneities [9]. The filtered phase images are scaled in the range of 0 to 1 by using a specified scaling function. By convention, a scaled phase of 1 represents a negative phase variation. The resulting phase mask is multiplied by the magnitude image on a voxel-by-voxel basis (Figure 3A) to form the final SWI. This process maximizes susceptibility contrast on SWI and even small vessels present very dark signals so that MRI venography can be obtained by applying a minimum intensity projection (MinIP) reconstruction into thicker slices.

QSM susceptibility values are calculated from phase images, which requires solving the ill-posed inverse problem (Figure 3B). This is achieved by imposing an approximation for the relationship between phase evolution and magnetic susceptibility distribution [29].

Since a direct inversion in the k-space is ill-conditioned due to regions of zero in the dipole kernel, alternative methods are needed for extracting the magnetic susceptibility. Most of these approaches use a minimization problem coupled with spatial regularizations to ensure consistency between tissue boundaries. However, QSM results are heavily dependent on the algorithm employed, and a consensus regarding the most suitable algorithm for all cases has yet to be reached [30]. Nonetheless, iterative methods demonstrate superior performance compared to both direct methods and current deep learning algorithms. Finally, QSM is a relative measure, which means that QSM values must be referenced. In practice, the reference region must be sufficiently large to mitigate inconsistencies and ideally exhibit minimal variation across subjects and scans. Frequently, the mean value of the whole brain is used as the reference. However, this strategy may not be suitable in the setting of neurological diseases when diffuse alterations in the tissue susceptibility are expected. As an alternative, the CSF can be used as a reference, whereby CSF values depend on the region of interest chosen [31], i.e., in the ventricle, it is important to avoid the regions of choroid plexus and calcification. Special QSM post-processing algorithms have been suggested to achieve minimal variation in cerebrospinal fluid (CSF) susceptibility for reference purposes [32].

**Figure 4:**
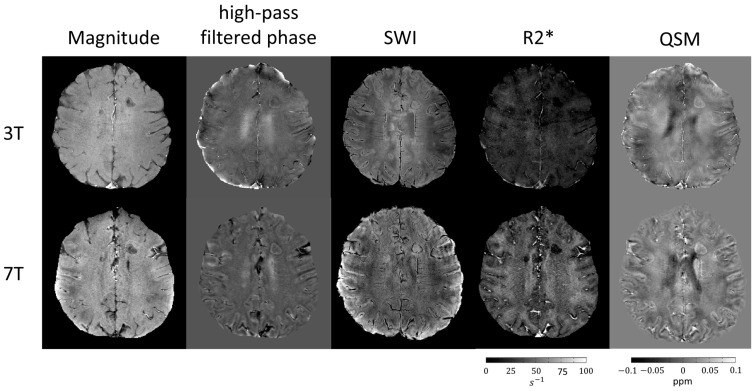
**MS lesion in different susceptibility imaging maps and acquired in different magnetic field strengths.** From left to right, magnitude image, phase image, SWI, R2* map, and QSM of an axial slice in an MS patient acquired at 3T (top row) and 7T scanner (bottom row). QSM was processed using the STISuite v 3.0 toolbox. The patient shows a large PRL in the left frontal white matter.

In terms of image acquisition, there are important differences between QSM and SWI. Both rely on fully flow-compensated 3D GRE sequences. However, while SWI requires the acquisition of only one echo (long TE), QSM requires a multi-echo acquisition, since the phase for different tissue types can vary over time [33]. With more echoes, the magnetic field shift can be estimated with more precision, and the signal–noise ratio (SNR) increases. The echo spacing should be uniform, the first echo as short as possible and the last echo at least equal to the T2* of the tissue of interest. Since QSM acquisition is based on a multi-echo GRE sequence, it is also possible to calculate R_2_* maps from the magnitude images obtained at different TEs. Figure 4 shows an example of the different image modalities based on susceptibility in an MS patient at 3- and 7T. All of them benefit from the stronger magnetic field strength at 7T, resulting in a higher susceptibility contrast. The higher magnetic field strength also offers a higher signal-to-noise ratio and a better spatial resolution at comparable acquisition times (Figure 4).

Albeit the more difficult implementation of QSM in clinical practice, there are definitely important advantages when compared to the other susceptibility techniques: it is quantitative, it does not suffer from blooming artifacts (augmentation in size of the susceptibility spot), and it distinguishes between diamagnetic and paramagnetic substances. R2* maps are quantitative, but they suffer from blooming artifacts, and they do not distinguish between paramagnetic and diamagnetic substances [34]. On SWI, both paramagnetic and diamagnetic compounds appear hypointense, and the only way to distinguish between them is to check their signal on the corresponding phase image. Phase images, on the other hand, are affected by the non-local susceptibility effects of adjacent tissues at a given point, which can end up mimicking false rims around lesions [35]. Among all the susceptibility-weighted techniques, QSM is the only one that by the deconvolution of the phase information infers the precise location of the underlying magnetic susceptibility sources, which can be determinant for the distinction between solid and shell MS lesions.

Another important difference between SWI and QSM is related to spatial resolution. In SWI, the main goal is the resulting contrast, therefore a common preference is to acquire 2–3 mm thick slices with a very high in-plane resolution of 0.2–0.4 mm. On the other hand, QSM requires a 3D convolution process which uses non-local information to compute the magnetic susceptibility of each voxel, therefore it is important to work with three-dimensional acquisitions and small isotropic voxels to avoid the underestimation of magnetic susceptibility [36]. In summary, while SWI represents a practical ready-to-use clinical tool, QSM demands longer acquisition times and sophisticated off-line post-processing. Nonetheless, both techniques are highly sensitive to magnetic sources in the tissue. Specifically, iron accumulation leads to increased magnetic susceptibility contrast in both SWI [9,11,37] and QSM [25,38,39], making these techniques extremely useful for the identification of paramagnetic rim lesions (PRLs). 

An important part of MS evaluation by MRI is to observe the effect of Gadolinium (Gd) contrast on MS lesions on T1-weighted images, since contrast enhancement reflecting BBB disruption can be interpreted as a marker of active neuroinflammation. The question is how Gd can affect MRI images based on susceptibility contrast. Gd injected in the blood can potentially decrease the T1 and T2/T2* relaxation times of water protons. The T2/T2* effect is driven by the Gd bolus passage and does not last very long (in the order of a couple of minutes) [40]. The T1 effect lasts much longer, but it highly depends on the distance between Gd ions and hydrogen protons (the closer the stronger the interaction); therefore, a significant T1 shortening is only visually appreciated on T1-weighted images in structures deprived of BBB or extra-axial compartments, such as dura matter, choroid plexus, blood vessels, or in lesions with BBB disruption. A study comparing SWI MRI signal before and after Gd injection at 1.5- and 3T observed a signal enhancement after Gd injection within veins, probably due to a “T1 shine through” effect [41], while there were no differences in the parenchymal regions of grey and white matter with preserved BBB [42].

### 2.3. Iron and Myelin on Susceptibility-Weighted Images

Inside a voxel, there is a mixture of substances giving rise to a net magnetic susceptibility χ.

Iron has a much higher susceptibility than other biological components. At the cellular level, iron is present in hemoglobin, neurons, astrocytes, microglia, and in oligodendrocyte cells [15,43], and can be found in two different ionic states, ferrous (Fe^2+^) and ferric (Fe^3+^) iron, with each of these states having different susceptibilities. It has been shown that reduction of ferric iron (5 unpaired electrons) to Fe^2+^ (only 4 unpaired electrons) results in decreased R2 and R2* values throughout the whole brain [44], with qualitative changes in susceptibility maps from QSM. This indicates that in addition to iron concentration, its oxidation state is also relevant for its contribution to the susceptibility-based contrast.

Myelin is diamagnetic, with a much lower absolute susceptibility compared to iron; however, its concentration in white matter is much higher, such that it becomes the main contributor of net magnetic susceptibility (χ) in white matter [21]. Furthermore, the magnetic susceptibility of myelin is also anisotropic [45], making the white matter signal sensitive to the head orientation in the scanner. In general, myelin fibers perpendicular to the main magnetic field present a more hypointense signal on QSM maps than parallel to it [46]. To ensure reproducibility, the placement of the head should be in the supine direction with the head straight relative to the z-direction of the scanner, with the readout direction set to the AC-PC line.

Histopathologic myelin content is positively correlated with R2* and inversely correlated with relative susceptibility (due to diamagnetic properties). Therefore, demyelination will have the opposite effect: a reduction in R2* and an increase in relative susceptibility. SWI hypointensities in MS lesions are considered to be mainly due to iron deposition within inflammatory cells and in a minor grade to demyelination, but to really disentangle both contributions within a voxel it is necessary to apply new algorithms that have been recently proposed to separate the diamagnetic from the paramagnetic component of the resulting measured susceptibility within a voxel [47,48].

### 2.4. Deoxyhemoglobin in Susceptibility-Weighted Images

The visualization of brain parenchymal veins in SWI relies upon the deoxyhemoglobin blood content. Hemoglobin is a molecule responsible for the oxygen transport in the body. It is composed of a protein matrix with four iron atoms in the ferrous state (Fe^2+^). When no oxygen is bound to the molecule (referred to as deoxyhemoglobin), the unpaired electrons from the iron atoms give rise to a paramagnetic behavior of the molecule as a whole [19]. For this reason, SWI represents a suitable tool for investigating venous blood. It should be noted though, that in MS the deoxyhemoglobin component in brain veins is often reduced when compared to healthy conditions [49], possibly due to the hypometabolism in the chronic phases of MS pathology [50], which leads to a lower MRI contrast of the veins on SWI images. The diminished vessel contrast can challenge the detection of CVS, especially at lower field strengths like 1.5T. Some authors demonstrated an advantage in injecting Gd for the detection of CVS in MS lesions [51,52].

When evaluating a CVS in MS, important factors are the lesion size and if the CVS is visible on more than one perpendicular plane. For this reason, the T2*-weighted image used to evaluate CVS should have preferentially a submillimetric and isotropic resolution. Since high-resolution 3D T2* GRE is time-consuming, an alternative is to use 3D T2* segmented echo planar imaging (EPI) sequences [53]. As opposed to the conventional single-shot EPI used for fMRI or perfusion studies, segmented EPI requires several shots (typically around 15 repetitions) to cover the whole k-space, i.e., the k-space is divided into more segments, which increases acquisition time compared to a single shot acquisition. However, it enables the acquisition of images with higher resolution than single-shot EPI, and in shorter times than 3D GRE sequences.

## 3. Application of Susceptibility-Weighted Techniques in Multiple Sclerosis

### 3.1. Central Vein Sign

In the last decade, the depiction of a central vein sign (CVS) within lesions became highly relevant for the differential diagnosis of MS [2]. It is associated with the fact that the visualization of a vein in the center of T2/FLAIR hyperintense lesions confirms the venocentric origin of a typical MS-lesion, which helps to differentiate other pathological changes, such as edema, gliosis, unspecific inflammation, and other conditions [1,54].

CVS was first described using T2*-gradient echo (T2*-GRE) [55] and SWI images acquired in 7T scanners. Shortly after, it was proven that CVS can be accurately assessed using 3T scanners [1,54]. The presence of a central vein is not exclusive to MS-lesions, as a vein can incidentally cross a brain lesion of different etiology [56], or because several inflammatory and vascular lesions can also grow around parenchymal vessels [57,58]. However, the CVS is visible in a significantly higher proportion of MS-lesions [56,59,60,61,62].

**Figure 5:**
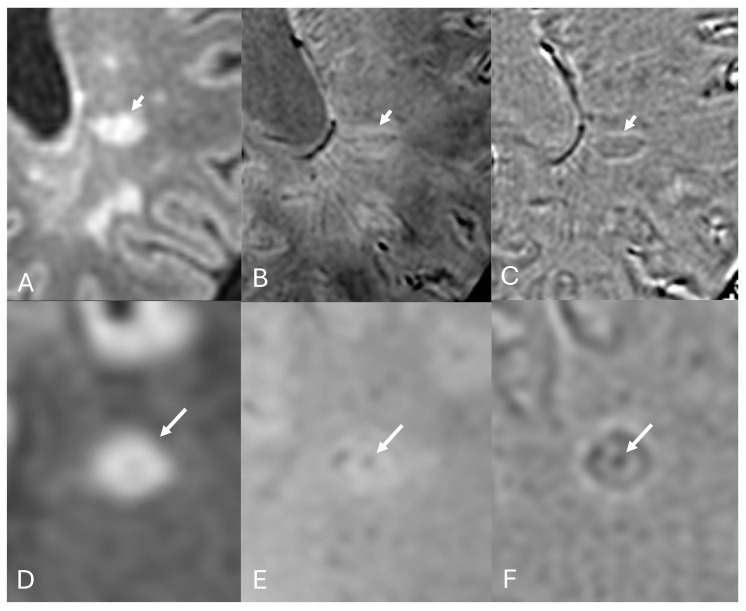
**Shapes of MS lesions with central veins.** When the MRI plane visualizes the lesion in the longest axis, and the lesion appears with an oval shape in FLAIR (arrow in (**A**)), the hypointense line of the central vein will be visible in SWI (arrow in (**B**)) and SWI-phase (arrow in (**C**)) crossing the center of the lesion and given a coffee-bean appearance. When the lesion appears with a round-shape in FLAIR (arrow in (**D**)), the central vein appears as a dark point in the center of the lesion in SWI (arrow in (**E**)), which will frequently have a target or doughnut-shape in SWI-phase (arrow in (**F**)).

The correct definition of the CVS follows precise radiological criteria and depends on the identification of certain properties on susceptibility-based MRI. First, to be eligible for evaluation, a lesion should be well-defined, have more than 3–5 mm in any diameter, and be non-confluent. The definition of a positive CVS requires the detection of a thin hypointense line or dot visible in at least two perpendicular planes. This vein should run partially or entirely through the lesion, equidistant from the lesion borders [57,63]. A typical MS-lesion with a CVS will appear as a coffee-bean or a doughnut, depending on the image plane [64] (Figure 5).

As CVS can eventually be present in non-MS lesions, it is necessary to establish specific cut-offs for determining MS-associated CVS. Previous studies have established thresholds based on the percentage of CVS+ lesions, varying from 29 to 54% [3,56,59,60,61,65,66]. While several studies adopt a cut-off of >40% CVS+ lesions to support the MS diagnostic, the applicability of this general rule remains limited. The frequency of CVS+ lesions varies across different diseases and is influenced by the specific imaging techniques employed [63].

Furthermore, the application of percentage-based approaches in routine radiological practice remains time-consuming. Recent efforts are being put into the development of automated and artificial intelligence methods to assess the CVS. Although some centers have developed tools with promising performance in detecting CVS and differentiating MS-lesions from its mimics [67,68], those tools still need to be tested and validated in larger and prospective cohorts.

Simplified criteria have been developed to enhance the practical applicability of the CVS in routine MS-diagnostic. These criteria rely on the count of three or six CVS lesions within a pool of brain lesions, with specific attention to characteristics such as shape, size, and location, ensuring eligibility for inclusion in the count. Notably, the count criteria have been applied in studies demonstrating sensitivity and specificity comparable to the percentage-based approaches [5,62,64,69,70]. The sensitivity of the central vein sign (CVS) ranges from 61.9% to 100%, while specificity varies from 73% to 100% across both percentage and count-based methodologies. The observed variability is contingent upon the chosen cut-offs, the methods for defining the central vein sign (CVS), and the characteristics of the studied cohort. It is crucial to note that most studies are retrospective [3,56,57,59,64,70] or have a restricted number of participants [60,61,66], introducing potential selection biases. Only a few recent investigations have adopted prospective designs with a more representative number of participants [5,62,69]. Therefore, larger prospective studies are still needed for establishing standardized protocols for the assessment of CVS in MS.

While ultra-high field MRI (7T and higher) allows an increased visualization of CVS+ lesions [71,72], the sensitivity and accuracy of central vein detection at 3T are comparable to 7T [73]. Given the wider availability of 3T compared to 7T, it is practical to utilize 3T for assessing CVS in MS diagnosis. On the other hand, given the lower sensitivity [74] in detecting parenchymal veins and the limited number of studies using 1.5T scanners [73], the usual cut-offs of 40 or 50% for CVS+ lesions may prove to be inadequate in distinguishing MS from its mimics, and this magnetic field strength might not be adequate for this purpose.

Different MRI techniques have been used to assess the CVS, including T2*-GRE, T2*-EPI, and SWI, usually employing 3D sequences. In 3T scanners, the sensitivity of these MRI modalities for detecting central veins ranks higher in the order of 3D T2*-EPI > 3D SWI > 3D T2*-GRE [73,75]. In 7T, studies have used 3DT2*-GRE, 3DT2*-EPI [76], and 3D-SWI with excellent characterization of parenchymal vascularization and central veins [55,71,73,77,78]. Nevertheless, all these techniques are suitable for evaluating CVS, with the quality and accuracy of the images contingent upon the researchers’ choices and adjustments of acquisition parameters.

Post-processing techniques, like FLAIR* (combining FLAIR with T2* [4,54,79] or SWI [78,80]), by adding cerebral spinal fluid-suppressed brain images, increase lesion-to-normal appearing white matter (NAWM) and NAWM-to-vein contrast. This improvement facilitates a better visualization of parenchymal brain structures and their relationship to central nervous system (CNS) lesions. Nevertheless, the use of FLAIR* does not significantly impact sensitivity in distinguishing MS from other diseases when compared to non-fused FLAIR and susceptibility-based images [73]. Another new post-processing technique, the susceptibility relaxation optimization, has demonstrated improved image quality, reduced artifacts, and enhanced CVS detection [81]. However, this method awaits testing in large and diverse cohorts and comparisons with other processing methods.

CVS primarily serves diagnostic purposes. However, the assessment of the frequency of the CVS+ lesions in radiologically isolated syndromes (RIS), and neurological syndromes suggestive of MS might not only discriminate MS from MS-mimics [5], but also play a possible role in stratifying and predicting the risk of conversion to clinically definite MS [82]. Additionally, it has shown a correlation with the extent of perivascular inflammation and demyelination in RIS-lesions [83], suggesting a potential, though not fully defined, prognostic value in these early syndromes.

It is still debatable whether CVS is solely a diagnostic tool or if the characteristics of veins within lesions or brain parenchyma can reveal other features of MS pathology severity. Few studies have indicated that in both MS and RIS, lesions with detectable central veins exhibit more pronounced signs of microstructural damage, as measured by diffusion MRI [79] and T2* relaxometry (R2*) [84]. This distinction proves valuable use in differentiating MS-lesions from comorbidity lesions (such as those seen in migraine and small vessel disease lesions) coexisting within the same MS patient [79], as well as discerning MS-related from MS-mimics in RIS patients [84]. Yet, unraveling whether the heightened tissue damage in CVS+ lesions merely signifies pronounced damage in any demyelinating lesion compared to other etiologies like small vessel pathology or migraine, or if, within MS lesions, the presence of a central vein also points to heightened surrounding microstructural damage remains a persistent challenge.

While one study observed a rather stable feature of CVS in MS-lesions follow-up [85], another study showed changes in venous diameter specifically in chronic active and shrinking lesions [86]. Additionally, a study using ultrasmall superparamagnetic iron oxide (USPIO), ferumoxytol, as a contrast agent, revealed both dilated vessels and increased vessel density within MS-lesions [80]. The pathological and prognostic meaning of those changes remains uncertain. Nevertheless, it is well established that immunological events linked to the formation of MS lesions typically induce BBB dysfunctions and are intricately associated with intra- and perivenular inflammation. An MRI-pathology study demonstrated venule remodeling and changes in the vessel wall progressing from inflammatory infiltrates to fibrinoid reactions with lesion age [77]. Although this study was partially based on the experimental models of autoimmune encephalomyelitis and a limited number of human post-mortem observations, it effectively showed that susceptibility-based MRI can capture dynamic changes in vessel-wall inflammation and fibrosis. While CVS is primarily employed as a diagnostic tool, this emerging evidence suggests that susceptibility-based sequences may detect evolutive changes in vascular wall and diameter, offering potential pathological, physiological, or prognostic insights.

### 3.2. Paramagnetic Rim Lesions

Multiple sclerosis pathological processes are triggered and, at least in the earliest stages of the disease, partially driven by acute inflammatory events highly associated with perivenular lymphocyte activity and BBB dysfunction. After about 10–15 years, a significant proportion of MS patients transition to a progressive type of the disease, with neurological deterioration (partially) independent from relapses and acute inflammatory activity, characterized by the accumulation of degenerative processes [87]. In recent years, a better understanding of pathological processes in MS has shown that relapsing and progressive phases are not dichotomous, but a result of continuous and concomitant inflammatory and degenerative processes, happening from the earliest MS stages [88].

The diagnosis of MS progression typically relies on a retrospective assessment, considering a patient’s history of gradual neurological deterioration. Currently, there are no specific MRI markers or criteria established for diagnosing progressive MS (PMS) [87]. These challenges are partially associated with the lack of understanding of the underlying pathological mechanisms behind PMS. Additionally, the conventional MRI assessments of MS heavily focus on detecting macroscopic lesion formation and inflammation linked to the BBB disruption [2].

One of the factors that is being recently associated with degenerative processes and disease progression in MS is the latent inflammatory activity found in chronic MS lesions, characterizing chronic active lesions [89,90,91]. This inflammatory activity in chronic active lesions is associated with the presence of iron-laden active microglia [10], visible in SWI images as a paramagnetic rim surrounding MS-plaques [10,11]. This distinctive feature defines what is known as PRLs [90,92], or iron-rim lesions (IRLs) [93,94,95].

Similar to the CVS, the PRLs were first seen and described in 7T-MRI [10,11,96,97]. Soon after, one study showed that 3T SWI-phase images detect a similar amount of paramagnetic rims in MS lesions when compared to 7T. Nevertheless, 3T images found more false-positive and false-negative lesions. One source of false-positive diagnoses was linked to a “rim-like” configuration of vessels around the lesion, which could induce dipolar fields and potentially interfere with the local susceptibility. This led to a discussion that rim evaluation in 3T should be careful in areas with a high density of vessels, such as the ventricular horns or the corpus callosum [37]. Only a few studies have been conducted using 1.5T scanners, and there is a scarcity of research comparing their sensitivity in paramagnetic-rim detection with 3T [98,99]. The paucity of data hinder the ability to draw conclusions regarding the reliability of studying this sign at lower MRI field strengths.

**Figure 6:**
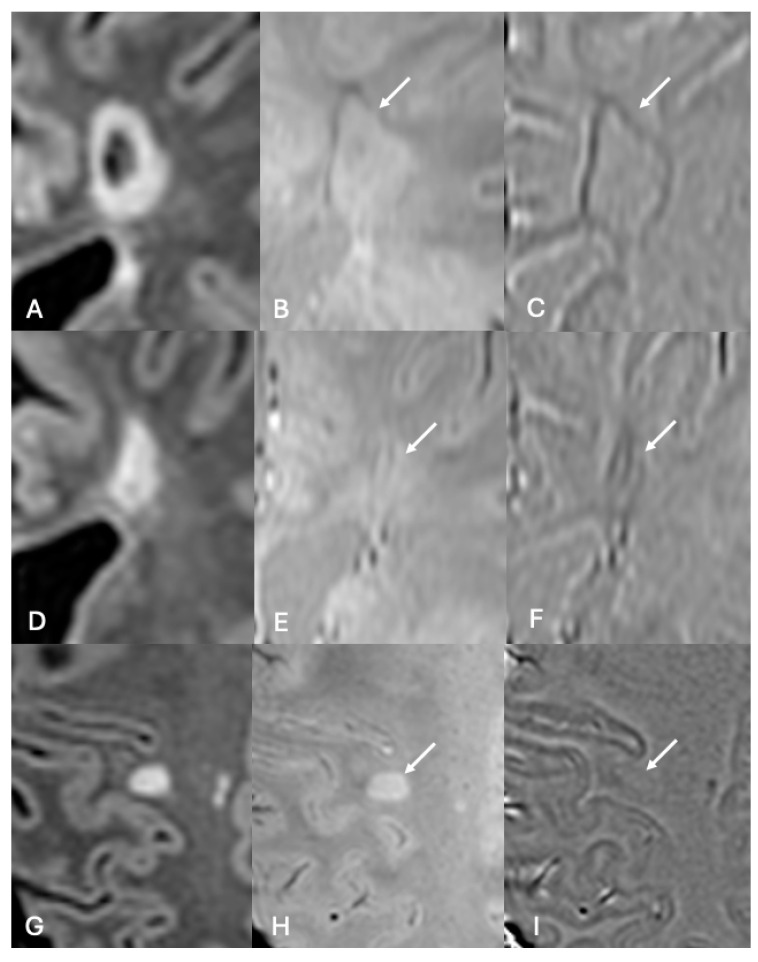
**Paramagnetic rim lesions in SWI and SWI-phase images.** The first FLAIR image (**A**) shows a cavitated MS plaque with an intense paramagnetic rim visible in both SWI (arrow in (**B**)) and SWI-phase (arrow in (**C**)) images. In another chronic active lesion (**D**), the paramagnetic rim is visible only in SWI-phase (arrow in (**F**)), but not in SWI (arrow in (**E**)), illustrating the higher sensitivity of SWI-phase in detecting paramagnetic rims. Similarly, in the bottom-down lesion (**G**), which appears to be a chronic inactive lesion in SWI (arrow in (**H**)), it is possible to visualize a fainty paramagnetic rim (**I**) in the SWI-phase map (arrow in (**I**)).

Regardless of the field strength, an adequate investigation of paramagnetic rims relies on acquiring high-resolution SWI/T2* images (submilimetric in-plane resolution) [11,37,100,101,102]. While the presence of a visible paramagnetic rim in T2*/SWI magnitude images indicates a pronounced susceptibility effect, SWI-phase images are more sensitive for studying PRLs and have been the preferred choice in most studies utilizing SWI [37,103] (Figure 6).

PRLs have a pivotal role in elucidating the underlying mechanisms of chronic inflammation and degeneration [39,94,104], and having a central role in studying disease progression. Still, the pathological mechanism responsible for the formation of paramagnetic rims seems to be specific to MS, occurring in a significantly higher frequency compared to brain lesions in other neurological diseases [65,90,92,105,106], suggesting a potential importance as a diagnostic marker. The detection of ≥1 PRL in an MRI has shown to be a highly specific sign to differentiate MS from other neurological diseases (specificity ranging from 93 to 99.7%) [90,92,105], but with a low sensitivity in most studies. The accuracy of the presence of PRLs increases when combined with the detection of CVS [90,105] and/or MS criteria for dissemination in space [65].

PRLs were virtually never described in association with non-inflammatory neurological diseases, such as cerebral small vessel disease and migraine [90]. Nevertheless, few studies have reported the presence of PRLs in some inflammatory and infectious neurological diseases, such as NMOSD [92,106], Susac syndrome, HTLV-associated myelopathy/tropical paraparesis [90], and one study has observed PRLs in diabetes mellitus [105]. It is still unknown whether the pathophysiological mechanisms underlying the paramagnetic rims in these diseases are similar to those in MS. Additional studies are required to unravel the histopathologic findings behind the rare occurrence of paramagnetic rims in non-MS diseases.

### 3.3. Iron Homeostasis, Demyelination, Remyelination, and Chronic Active Lesions

Iron is an essential cofactor of several cell reactions, having an important physiological role in myelin synthesis and maturation, neurotransmission, and oxygen transport and ATP production [107]. In the brain, the oligodendrocytes have the highest concentration of iron, and the maturation process of oligodendrocyte progenitor cells (OPCs) is highly dependent on iron intake and accumulation [15]. Nevertheless, iron dyshomeostasis is detrimental to the CNS, being involved in several pro-inflammatory and degenerative cascades [13,89].

During the formation of a demyelinating plaque, there is an abrupt release of iron in extracellular spaces. Unbound ferrous iron (Fe^2+^) is toxic and catalyzes the production of reactive oxygen species (ROS) and must be phagocytized by brain macrophages/microglia [89]. In order to achieve iron homeostasis, iron ions have to be reduced to Fe^3+^ and stored as ferritin and, to induce remyelination, a sufficient amount of iron has to be donated to OPCs and oligodendrocytes [15].

The factors contributing to a prolonged time to remyelinate or a remyelination failure, resulting in the persistence of chronic active lesions (CALs) or slowly expanding lesions (SELs), remain elusive. However, the analysis of (dynamic) histopathological and neurochemical changes in demyelinating lesions allows some insights. The persistence of chronic inflammation and microglial activation at lesion margins is more frequent in larger demyelinating plaques [11,108]. The lesions with persistent paramagnetic rims tend to have longer T1 relaxation times [97], very low R2* [39], lower myelin water fractions [24], and higher apparent diffusion coefficient at the lesion center [109], reflecting more pronounced demyelination and tissue destruction.

Macrophages in the CNS exist in three distinct states: non-polarized macrophages (M0), proinflammatory microglia (M1), and phagocytic macrophages (M2). Increased iron uptake by macrophages favors M1 polarization, increasing the release of TNF-α and ROS [10]. This M1 pro-inflammatory cycle is a dose-dependent response to the abnormal iron release from oligodendrocyte death, suggesting that larger lesions with more pronounced acute inflammation [108] and demyelination are more likely to induce excessive M1 polarization, perpetuating the inflammatory and cytolytic cycle.

In the periphery of a demyelinating lesion, iron can be uptaken not only by activated microglia but also by astrocytes. Under non-pathological conditions, astrocytes play a central role in exporting iron across the BBB and from the brain tissue to both oligodendrocyte precursor cells (OPCs) and mature oligodendrocytes [15], thereby contributing to myelin maturation, maintenance, and repair. Astrocytes produce high levels of glutathione, a potent ROS scavenger. This capability potentially protects the tissue from oxidative stress and makes astrocytes more resistant to iron overload [89].

Astrocytes also play a role in trafficking iron from and to microglia cells [15]. A beneficial remyelinating response entails an orchestrated interaction between macrophages/microglia and astrocytes. This intricate process includes the phagocytosis of demyelinating debris, clearance of free iron, secure storage of iron as ferritin in intracellular compartments, and the eventual release of iron to OPCs. Prolonged iron overload in astrocytes and microglia may surpass their capacity to convert iron to ferritin, leading to an excessive generation of ROS and oxidative damage, ultimately resulting in increased cellular damage [10]. Moreover, in the setting of chronic demyelination and abnormal glial scar formation, the migration of oligodendrocyte precursor cells (OPCs) to demyelinated areas is hindered. Instead of being efficiently recycled to remyelinating cells, the iron released from dying cells is directed toward pro-inflammatory cells, exacerbating oxidative stress [13]. While the mechanisms impeding chronic inflammation, demyelination, and persistent tissue destruction are not fully understood, it appears that the failure to establish iron homeostasis plays a central role.

### 3.4. Persistent Paramagnetic Rim and Slowly Expanding Lesions

There is increasing attention to the presence of paramagnetic/iron rims in chronic MS lesions as a sign of latent inflammation [10,97], persistent demyelination [11,107], and increased risk of brain degeneration [94,104] and disease progression [12,91]. However, dynamic changes in iron distribution and compartmentalization are present in all stages of MS pathology, from lesion formation to remyelination, and persistent degenerative processes. In hyperacute demyelinating lesions, paramagnetic rims can appear or gradually increase shortly after the appearance of gadolinium enhancement [11] in up to 81% of the Gd+ lesions [102], or appear after the contrast enhancement subsides [11,110]. The delayed appearance of paramagnetic rims could be attributed to a predominance of M2 macrophage polarization during the hyperacute phase of demyelination. These myelin-laden cells exhibit a diminished capacity for iron uptake [10,110].

A proportion of these acute PRLs persist over time. The persistence of paramagnetic rims for longer than 12 months is associated with less shrinkage or lesion growth, lower T1 relaxation times, and the progressive increase of apparent diffusion coefficient (ADC) values [11,109], reflecting more pronounced and persistent tissue destruction. After their appearance, the iron rims of PRLs tend to stay stable in the first 5 years, gradually decreasing after this time [93]. However, more longitudinal studies are needed to confirm this observation and establish the average/maximum duration of iron rims.

The recognition of paramagnetic rim lesions (PRLs) as potential risk factors for long-term disability progression and intensified brain degeneration, as evidenced by elevated rates of brain atrophy [94,111] and increased levels of specific fluid biomarkers such as serum neurofilaments (sNFLs) [104], has led to the expectation that a higher prevalence of PRLs would be observed in progressive MS. Initial pathological investigations have indeed indicated a significantly higher prevalence of chronic active lesions featuring a rim of activated microglia in demyelinated areas in progressive MS compared to RRMS [112,113]. However, in vivo studies present conflicting findings, with some demonstrating a greater occurrence or proportion of PRLs in progressive MS [12,90,91,114], while others report a higher number of PRLs in RRMS [93,115]. Nevertheless, regardless of the disease subtype, all studies show that PRLs are associated with higher disability [114] or disability progression, higher relapse rates, cognitive decline [91,114,116], and other signs of a poor outcome.

The presence of paramagnetic rims has been consistently associated with slowly expanding lesions (SELs) in previous studies, which have also shown that lesions with these rims tend to exhibit greater growth rates over time compared to those without [93,97,109]. However, it is important to acknowledge that while PRLs are commonly associated with SELs, not all SELs necessarily display peripheral paramagnetic rims, as evidenced by recent studies [93,100]. The reasons behind continuous lesion growth in the absence of detectable paramagnetic rims remain unclear, although several hypotheses could be considered.

Firstly, chronic active inflammation might serve as a significant trigger for persistent demyelination and perilesional degenerative processes, which could endure even after the resolution of the iron imbalance. Secondly, SELs may be associated with demyelinating and degenerative processes independent of microglia activation [13,100]. Thirdly, it is also possible that the MRI methods used to depict the paramagnetic rims lack the sensitivity or specificity required to capture complex changes in MS plaques. For instance, SWI and QSM are techniques capable of non-invasively accessing changes in tissue susceptibility. However, demyelinating lesions can present a mixture of paramagnetic and diamagnetic sources [48]. In this way, ongoing dynamic changes in the diamagnetic components of myelin could obscure the presence of paramagnetic iron in activated microglia.

Other important factors that may contribute to the dissociation between PRLs and SELs are age- and disease-related variables occurring within MS plaques, perilesional areas, and normal-appearing tissues. Previous studies have shown that PRLs present higher WM FLAIR signal, lower T1 relaxation times [115,117], and higher mean diffusivity (MD) and mean kurtosis (MK) [118] in periplaque tissues, reflecting more pronounced microstructural damage outside of lesions. Additionally, studies have shown a decrease in iron lesions and overall iron content in normal-appearing tissues in the brain decreasing with age [13]. Although iron imbalance is an important factor contributing to the perpetuation of inflammatory processes and oxidative damage, it is also crucial for promoting remyelination. In this context, chronic inflammation in earlier stages of the disease, prolonged disease duration, and intrinsic changes in tissue iron content related to aging may deplete the repairing capacity of the tissue.

## 4. Conclusions and Future Directions

Susceptibility-weighted images encompass a set of MRI techniques that provide crucial tools for advanced MS diagnosis and prognostic evaluation, including two recently described indicators: the CVS and PRLs. SWI venography facilitates detailed visualization of intraparenchymal venous anatomy and its association with brain lesions, enabling the precise assessment of lesion morphology. This aids in early MS diagnosis and differential diagnosis. The ability to depict intricate changes in tissue susceptibility offers a valuable non-invasive and in vivo tool for assessing the dynamics of demyelination, remyelination processes, and their relationship with iron metabolism.

Regarding CVS, future studies should focus on the validation of CVS for diagnostics, establishing ideal protocols and recommendations for its routine use in MS diagnostics. Also, it is important to create and validate automatic methods for the detection and segmentation of CVS+ lesions using artificial intelligence. It is still unclear the relevance of dynamic changes in the venous caliper and perivenous pathology to MS progression and lesion and periplaque microstructural tissue destruction. So, the improvement of techniques to visualize intraparenchymal veins by using ultrasmall superparamagnetic iron oxide (USPIO), high-resolution MRI and/or ultra-high-field scanners can add insights to this point.

Recent studies have underscored the relevance of PRLs to the study of chronic active lesions and latent tissue inflammation independent of BBB disruption. However, further prospective longitudinal studies in larger cohorts are imperative to a better understanding of the PRL dynamics across MS stages and subtypes, giving a more comprehensive understanding of the association between PRLs and SELs and their impact on the progressive stages of MS. Moreover, the development of new MRI acquisition and processing is essential for untangling the components of demyelination and iron accumulation within lesion margins. Table 2 summarizes the main MRI signs and features derived from SWI, their limitations, and ongoing questions and some future directions for research in the field.

## Figures and Tables

**Table 1:** Susceptibility values for some compounds, brain structures in healthy brains, and MS lesions.

Compound/Structure	Magnetic Susceptibility (ppm)	Reference
Water (at 37 °C)	−9.04	Arrighini et al., 1968 [17]
Phopholipids *	−9.68	Kawamura et al., 1981 [18]
Lipids	−10.0	Schenck, 1992 [19]
Cortical bone	−8.7	Schenck, 1992 [19]
Choroid plexus	−0.14	Oshima et al., 2020 [20]
Calcified lesions	−0.26	Oshima et al., 2020 [20]
Deoxyhemoglobin molecule	0.2	Schenck, 1992 [19]
Red blood cells (deoxygenated)	−6.52	
Deoxygenated blood (Hct = 0.45)	−7.9	
Ferritin molecule (4500 iron atoms)	520	
Brain Tissue in vivo (relative to frontal deep WM) **		
Caudate nucleus	0.044	Deistung et al., 2013 [22]
Putamen	0.038	
Red nucleus	0.100	
Substantia nigra	0.111	
Globus pallidus	0.131	
Gray matter	0.020	
White matter	−0.030	
MS lesions (relative to CSF) *		
Rim+ lesion	0.006/0.002	Yao et al., 2018 [24] Kaunzner et al., 2019 [25]
Rim− lesion	−0.007/−0.015	
Susceptibility at the rim in rim+ lesions	0.013/0.020	
Susceptibility at the core of rim+ lesions	0.006/0.005	

* mean value, ** values obtained through QSM, abbreviations: CSF: cerebral spinal fluid; Hct: hematocrit; MS: multiple sclerosis; WM: white matter.

**Table 2:** Summary of novel MRI signs, the features of MS lesions and pathology, and the use of susceptibility-weighted images and longitudinal evaluation of chronic MS plaques.

	Central Vein Sign (CVS)	Paramagnetic Rim Lesion (PRLs)
Definition	Presence of a vein in the center of an MS plaque, visible in T2*/SWI image Lesion requisites for CVS confirmation: - Well-defined isolated/non-confluent lesions - Vein visible as a dark dot or line equidistant to the lesion margins - The visualization of CVS on more than one orthogonal plane	Paramagnetic rim around an MS lesion - Dark line in T2*/SWI magnitude images - Dark or bright line in SWI-phase (depending on the direction/polarity of the main static magnetic field) - Bright line in R2*
Pathological meaning	Captures the venocentric growth of MS lesions	Paramagnetic rim = mainly driven by iron-laden macrophages (activated microglia). Demyelination also increases the local susceptibility of the tissue and can collaborate with the paramagnetic effect
Main application in MS	Advanced diagnostic - Increase sensitivity and specificity - Potential inclusion in diagnostic criteria to improve early and precise diagnostic - Differential diagnostic with MS MIMICS	Prognostic tool - The presence of persistent PRL+ = higher risk of disability, MS progression, and brain degeneration - PRL+ = chronic active MS lesion Differential diagnostic - PRLs show high specificity for MS
Secondary applications	Prognostic tool - Changes in central vein diameter might reflect different inflammatory stages - Microstructural changes in MS lesions with central vein might reflect different degrees of tissue destruction	- The investigation of pathological mechanisms in MS and neuroinflammatory diseases - The investigation of new therapeutic targets
Limitations	- To count all CVS+ is time-consuming - The lack of uniform diagnostic criteria for all MRI field strengths, specially 1.5T - The visualization of changes in vein diameter is still challenging	- SWI might produce artifacts related to head position, microstructural changes, and peripheral veins (which can be minimized in 7T or by using QSM) - Iron and demyelination produce similar paramagnetic effects, being difficult to disentangle in the traditional approach of susceptibility-weighted techniques
Future directions	- The development and validation of artificial intelligence/automatic tools for CVS detection and count - The improvement of the visualization of CVS/dynamic changes in venous diameter: ○ The use of ultrasmall superparamagnetic iron oxide (USPIO) ○ High-resolution and/or ultra-high field MRI	- Longitudinal studies: define changes in PRLs in different MS subtypes and the relationship between PRLs and slowly expanding lesions (SELs) - The development/application of new MRI acquisitions and post-processing techniques to separate the signal of different paramagnetic compounds in PRLs (i.e., demyelination and iron)
